# Fetal DNA Methylation Associates with Early Spontaneous Preterm Birth and Gestational Age

**DOI:** 10.1371/journal.pone.0067489

**Published:** 2013-06-27

**Authors:** Sasha E. Parets, Karen N. Conneely, Varun Kilaru, Stephen J. Fortunato, Tariq Ali Syed, George Saade, Alicia K. Smith, Ramkumar Menon

**Affiliations:** 1 Genetics and Molecular Biology Program, Emory University, Atlanta, Georgia, United States of America; 2 Department of Human Genetics, Emory University School of Medicine, Atlanta, Georgia, United States of America; 3 Department of Psychiatry & Behavioral Sciences, Emory University School of Medicine, Atlanta, Georgia, United States of America; 4 The Perinatal Research Center, Nashville, Tennessee, United States of America; 5 Department of Obstetrics & Gynecology, Division of Maternal-Fetal Medicine Perinatal Research, The University of Texas Medical Branch at Galveston, Galveston, Texas, United States of America; John Hunter Hospital, Australia

## Abstract

Spontaneous preterm birth (PTB, <37 weeks gestation) is a major public health concern, and children born preterm have a higher risk of morbidity and mortality throughout their lives. Recent studies suggest that fetal DNA methylation of several genes varies across a range of gestational ages (GA), but it is not yet clear if fetal epigenetic changes associate with PTB. The objective of this study is to interrogate methylation patterns across the genome in fetal leukocyte DNA from African Americans with early PTB (24^1/7^–34^0/7^ weeks; N = 22) or term births (39^0/7^–40^6/7^weeks; N = 28) and to evaluate the association of each CpG site with PTB and GA. DNA methylation was assessed across the genome with the HumanMethylation450 BeadChip. For each individual sample and CpG site, the proportion of DNA methylation was estimated. The associations between methylation and PTB or GA were evaluated by fitting a separate linear model for each CpG site, adjusting for relevant covariates. Overall, 29 CpG sites associated with PTB (FDR<.05; 5.7×10^−10^<p<2.9×10^−6^) independent of GA. Also, 9637 sites associated with GA (FDR<.05; 9.5×10^−16^<p<1.0×10^−3^), with 61.8% decreasing in methylation with shorter GA. GA-associated CpG sites were depleted in the CpG islands of their respective genes (p<2.2×10^−16^). Gene set enrichment analysis (GSEA) supported enrichment of GA-associated CpG sites in genes that play a role in embryonic development as well as the extracellular matrix. Additionally, this study replicated the association of several CpG sites associated with gestational age in other studies (*CRHBP*, *PIK3CD* and *AVP*). Dramatic differences in fetal DNA methylation are evident in fetuses born preterm versus at term, and the patterns established at birth may provide insight into the long-term consequences associated with PTB.

## Introduction

Despite advances in health care, the rate of preterm birth (PTB; birth before 37 weeks of gestation) has been increasing for the last 25 years [1]. Specifically, children born preterm are more likely be hospitalized and have diminished cognitive performance and develop behavioral problems such as ADHD during childhood [2], [3]. Along these lines, many adult onset diseases have been linked to adverse intrauterine conditions or adverse pregnancy outcomes [4], [5]. Thus, PTB not only imparts a difficult start but also considerable challenges throughout life [1], [6]. Spontaneous preterm birth (PTB), which occurs without indications, is common and contributes to significant neonatal morbidity and mortality over time [7].

Several epidemiologic, behavioral and biological factors (i.e. race, socioeconomic status, malnutrition, smoking, and infection) have been associated with PTB, but the mechanistic pathways that underlie the association of the risk factors to PTB are still unclear [8], [9], [10]. The field of epigenetics has the potential to provide a greater understanding of the pathways that contribute to or result from PTB [11]. Indeed specific risk factors may promote epigenetic changes that result in PTB or that predisposes a neonate to adult-onset diseases. Although epigenetic differences associate with many prenatal exposures and complex traits, published studies that evaluate maternal and fetal epigenetic changes during pregnancy, influence on pregnancy outcome, and fetal programming of adult-onset diseases are limited [12], [13]. The study of epigenetic patterns during early development is likely to provide more information about environmental and behavioral influences on long-term outcomes than the study of individuals later in life. In time, such studies may suggest biomarkers for developmental outcomes.

DNA methylation is an epigenetic modification required for proper gene regulation and cellular differentiation during fetal development [14], [15]. Over the first years of life, DNA methylation of many genes appears to be relatively stable [16], [17]. Therefore, DNA methylation patterns of certain genes established at birth may result in a developmental trajectory with long-term consequences. We have previously shown that DNA methylation of certain genes associates with gestational age (GA) in term deliveries [18], and evidence suggests that DNA methylation differences in key genes may provide insight into biological pathways that underlie PTB. The primary objective of this study is to interrogate methylation patterns across the genome in DNA derived from umbilical cord blood leukocytes of a high risk African American cohort and to evaluate the association of each CpG site with PTB and GA.

## Methods

This study was approved by the Institutional Review Boards of Centennial Women’s Hospital, Western Institutional Review Board and the University of Texas Medical Branch.

### Subjects and Sample Collection

*The Nashville Birth Cohort (NBC*) was established to examine genetic risk factors and changes in the biochemical pathways that distinguish spontaneous preterm from term labor. All subjects were recruited at Centennial Women’s Hospital and the Perinatal Research Center in Nashville, TN beginning in 2003. Pregnant women were enrolled during their first clinical visit after obtaining informed consent. Maternal demographic and clinical data were recorded from medical records or by interviews during the consenting process. Demographic and clinical data specific to the fetus was collected from clinical records. Gestational age of the neonate was determined by maternal reporting of the last menstrual period and corroboration by ultrasound dating. Race was identified by self-reporting that traced back to three generations from maternal and paternal side of the fetus. Only African Americans of non-Hispanic ethnicity were included in this study.

Subjects were included in this study if they had contractions (rate of 2 contractions/10 minutes) leading to delivery either at preterm or term. Cases were delivered preterm with intact membranes between 24^1/7^ weeks and 34^0/7^ weeks. Controls were delivered (>39^0/7^ weeks) with spontaneous term labor and delivery and no current or history of pregnancy-related complications including PTB and preterm or prelabor rupture of the membranes (pPROM). Subjects who had multiple gestations, preeclampsia, placenta-previa, fetal anomalies, and/or medical or surgical complications during pregnancy were excluded from the study. Subjects with any surgical procedures during pregnancy were treated for preterm labor or for suspected intra-amniotic infection and delivered at term were excluded from the control group. Maternal demographic and clinical data were collected from medical records or thorough self-report at the time of consent.

Race, socioeconomic (education, household income, marital status, and insurance status), behavioral (cigarette smoking) factors were documented by maternal self-report. Intraamniotic infection was determined by amniotic fluid culture or by PCR for 16 s ribosomal RNA. In cases where culture or PCR data were not available, infection was assessed with four of the following clinical or histologic symptoms: high fever (>102°C), high CRP (>0.8 U/ml), abdominal tenderness, fetal tachycardia, mucopurulent vaginal discharge or histologic chorioamnionitis, funisitis.

### Biological Sample Collection and DNA Extraction

Umbilical cord blood samples were collected in EDTA tubes soon after placental delivery. Blood samples were centrifuged at 3,000 RPM to separate plasma, and buffy coats were aliquoted and stored at −80°C. DNA was extracted using the Autopure automated system (Gentra Systems, Minneapolis, MN).

### DNA Methylation Analysis

For each subject, >485,000 CpG sites across the genome were interrogated using the HumanMethylation450 BeadChip (Illumina, San Diego, CA) [19], [20]. Briefly, 1 ug of DNA was converted with sodium bisulfite, amplified, fragmented, and hybridized on the HumanMethylation450 BeadChip (Illumina, San Diego, CA) according to the manufacturer’s instructions. CpGassoc [21] was used to perform quality control and calculate ß values. Data points with probe detection p-values >.001 were set to missing, and CpG sites with missing data for >10% of samples were excluded from analysis; 483,830 CpG sites passed the above criteria. Samples with probe detection call rates <90% and those with an average intensity value of either <50% of the experiment-wide sample mean or <2,000 arbitrary units (AU) were excluded from further analysis. One sample of male DNA was included on each BeadChip as a technical control throughout the experiment and assessed for reproducibility using the Pearson correlation coefficient, to ensure that Pearson correlation coefficient >0.99 for all pairwise comparisons of technical replicates. For each individual sample and CpG site, the signals from methylated (M) and unmethylated (U) bead types were used to calculate a beta value as ß = M/(U+M).

### Statistical Analysis

We used MethLAB [22] to test for association with PTB via linear regressions that modeled β-values as the outcome and PTB as the independent variable, adjusting for GA, gender, chip, and row on the chip. Based on previous reports and the potential contribution to PTB we examined the association of birth weight percentile, gravidity, parity, infection and smoking as confounding factors in our analysis; these factors did not associate with methylation of any CpG site after adjustment for multiple testing (FDR<.05; data not shown). Birth weight percentile was based on estimated gestational age (GA) in accordance with the United States national registry [23]. We subsequently used MethLAB to fit similar linear regressions that modeled GA as the independent variable, adjusting for gender, chip, and row on the chip. Because it has been suggested that logit-transformed β values (a.k.a. M values) may perform better in statistical analyses [24], we also examined associations with M values using the strategy described above. Because there was no significant difference between the results, we present results based on untransformed β to ease biological interpretation.

The location of each CpG site was determined using the Illumina array annotation for the HumanMethylation450 BeadChip based on build 37 of the human genome. We tested for enrichment among GA-associated sites by comparing the number of GA-associated CpG sites that did or did not occur in a particular gene region (e.g. promoter, 5′UTR, Body, 1^st^ exon, 3′UTR, or intragenic regions) to the number of non-GA-associated sites that did or did not occur in that gene region, using Fisher’s exact test. We then performed similar tests of enrichment for CpG-rich regions defined as islands or CpG poor regions defined as shores [25], [26]. CpG sites with 1000 Genomes Project variants physically contained within the Illumina probe were noted in the analyses but not excluded a priori. In addition we examined whether significant GA-associated CpG sites were enriched or depleted on the X chromosome using Fisher’s exact test.

We used GSEAPrerank [27], [28] to evaluate whether GA-associated CpG sites were located in genes that were enriched for specific biological processes and cellular components. Significance of the gene ontology enrichment was corrected for an FDR<.05 following 1000 permutations.

## Results

The cohort, described in Table 1, consists of African American preterm (GA range 24.1–34.0 weeks) and term (39.0–40.9 weeks) births. Though the groups differed by GA and birthweight, they did not differ significantly in demographic or clinical factors.

**Table 1:** Clinical and demographic characteristics of the cohort.

Phenotype	PTB (N = 22)	Term Birth (N = 28)	p-value*
	Mean±SD	Mean +/− SD	
Male, %	(14) 63.6%	(11) 39.3%	NS
Gestational age, weeks	30.8±3.3	39.8±0.4	<.0001
Birthweight, grams	1524.1±638.1	3304.9±333.4	<.0001
Birthweight percentile	32±27.7	47±25.3	NS
Gravidity	2.2±1.5	2.4±2.7	NS
Maternal Age	25.5±5.2	21.0±4.6	NS
Employed	(5) 22.7%	(8) 36.3%	NS
Married	(7) 35.0%	(7) 25.0%	NS
Maternal Smoking	(5) 22.7%	(5) 17.9%	NS
Income			
<15 K	(12) 54.5%	(12) 42.9%	
15–30 K	(5) 22.7%	(8) 28.5%	NS
>30 K	(5) 22.7%	(8) 28.5%	

All subjects are African American.

* NS indicates the p-value is not significant (p>.05).

### Preterm Birth (PTB)

**Figure 1:**
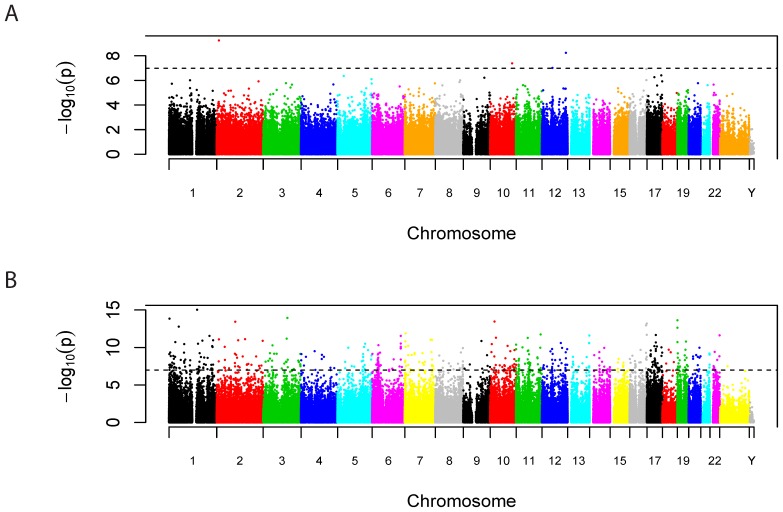
Manhattan plots depicting the association of all CpG sites with (A) PTB and with (B) GA. The y-axis is the negative log10 of the p-value for the association while the x-axis is position on each chromosome. The dashed line indicates experiment-wide significance. Genomic locations of each CpG site are in Table S1.

After accounting for multiple comparisons (FDR<.05) and confounding factors (gender, gestational age, and chip effects), 29 CpG sites associate with PTB independently of GA (Figure 1A; Table 2; 5.7×10^−10^<p<2.9×10^−6^;−.17<Δβ<.26). Based on annotation with data from the 1000 Genomes Project, 5 of these 29 CpG probes (17.2%) do contain a SNP (estimated average minor allele frequency of 15.5%), suggesting that we could be observing a genetic rather than an epigenetic association for these 5 CpG sites; the methylated and unmethylated signals for these five sites are shown in Figure S1. In some cases, the pattern appears consistent with SNP-induced methylation differences, while in other cases there is no strong pattern of clustering. Results were not significantly altered by adjustment for maternal smoking, or infection, birth weight percentile, and gravidity (data not shown) nor were they altered by logit-transformation of the beta values. Among the CpG sites associated with PTB, we observed increased DNA methylation of a site (cg13250001) in *GSK3B* (glycogen synthase kinase 3 beta; p = 1.7×10^−6^; Δβ = −.06) and decreased methylation of a CpG site (cg25376491) in *MAML1* (mastermind-like 1; p = 1.8×10^−6^; Δβ = .14) in fetuses with PTB. In addition, 3 other CpG sites in GSK3B and 4 in MAML1 were nominally associated with PTB (p<.05).

**Table 2:** 29 CpG sites that associated with PTB (adjusted for GA).

Probe ID	Gene	Δβ	t-statistic	p-value
cg04549583		−0.133	−8.30	5.70×10^−10^
cg13290254		0.122	7.52	5.77×10^−9^
cg03254336		0.178	6.87	4.24×10^−8^
cg16447680	*KIAA0748*	−0.131	−6.60	9.84×10^−8^
cg03272932		0.175	6.54	1.19×10^−7^
cg03152187	*SEPT9*	−0.112	−6.15	3.91×10^−7^
cg18721397	*SUB1*	0.259	6.12	4.31×10^−7^
cg03706951		−0.142	−6.04	5.57×10^−7^
cg13514049		0.129	6.01	6.13×10^−7^
cg01142526	*N4BP3*	−0.049	−5.92	8.00×10^−7^
cg03901454		0.174	5.86	9.65×10^−7^
cg19787650	*FAM49B*	0.181	5.85	9.88×10^−7^
cg20253872	*AMPD2*	−0.174	−5.85	1.00×10^−6^
cg06320380	*TNS1*	−0.111	−5.79	1.20×10^−6^
cg23471393		0.185	5.79	1.23×10^−6^
cg26501007		0.135	5.75	1.38×10^−6^
cg13250001	*GSK3B*	−0.062	−5.68	1.70×10^−6^
cg20519581		0.224	5.68	1.73×10^−6^
cg04212285	*PTPRN2*	0.155	5.67	1.75×10^−6^
cg25376491	*MAML1*	0.14	5.66	1.82×10^−6^
cg00101629	*KAZN*	0.147	5.65	1.86×10^−6^
cg19921917	*PALLD*	0.177	5.61	2.15×10^−6^
cg12207930	*MED12L*	0.198	5.59	2.24×10^−6^
cg10131972	*TXNRD2*	−0.077	−5.59	2.25×10^−6^
cg09964921		0.162	5.57	2.44×10^−6^
cg01476222	*TRAF6*	0.144	5.56	2.49×10^−6^
cg03318906	*RAB11FIP1*	0.128	5.54	2.60×10^−6^
cg01621943		0.185	5.54	2.65×10^−6^
cg13749927	*DDB2*	−0.070	−5.51	2.91×10^−6^

Δβ represents the average methylation difference between early PTB and term birth after adjustment for covariates.

### Gestational Age

Our above analyses of PTB all included GA as a covariate because PTB and GA are by definition correlated (r = .93), and there is overwhelming agreement in the association of DNA methylation with PTB unadjusted for GA, or GA itself (Figure S2). In fact, 9637 CpG sites associated with GA independent of gender and chip effects (FDR<.05; 9.5×10^−16^<p<1.0×10^−3^; −.024<Δβ per week<.023; Figure 1B: Table S1). GA-associated CpG sites were depleted in the promoter, first exon and 3′UTR regions and enriched in the 5′UTR, gene body and intragenic regions (2.2×10^−16^<p<2.6×10^−3^; Table 3) when compared to CpG sites that were not associated with GA via Fisher’s exact test. Associated CpG sites were also depleted in CpG islands (14.9% vs. 31.3%; p<2.2×10^−16^) and enriched in CpG shores (34.1% vs. 22.8%; p<2.2×10^−16^). Examining the directionality of GA-associated CpG sites, 61.8% (5958 CpG sites) had lower methylation in subjects with lower GA; these CpG sites were twice as likely to be located in CpG islands (p<2.2×10^−16^; Table 3) and less likely to occur in the gene body (p<2.2×10^−16^;) and 3′UTR (p = 1.5×10^−9^). While the sample size was not sufficient to look for sex-specific differences (i.e. interactions between age and sex), we did note a depletion of GA-associated CpG sites on the X chromosome (.5% vs. 2.4%; p<2.2×10^−16^); both the depletion of GA-associated variants on CpG islands and the X chromosome are consistent with a previous report of age-associated methylation in children [29].

**Table 3:** Enrichment analysis to examine whether there is a an enrichment in certain regions that associated with GA, or whether there is an enrichment of a certain direction of a t-statistic for associated CpG sites.

	GA-associated	Not GA-associated	p-value	(+) GA-associated	(−) GA-associated	p-value*
CpG Islands	14.9%	31.3%	<2.2×10^−16^	18.4%	9.4%	<2.2×10^−16^
CpG Shores	34.1%	22.8%	<2.2×10^−16^	33.4%	35.2%	NS
Promoter	22.9%	25.0%	2.5×10^−6^	25.3%	18.7%	4.7×10^−14^
5′ UTR	10.1%	8.8%	6.0×10^−6^	8.6%	12.5%	1.7×10^−9^
1st Exon	3.1%	4.7%	4.7×10^−15^	3.0%	3.3%	NS
Gene Body	35.0%	33.3%	4.4×10^−4^	29.6%	43.7%	<2.2×10^−16^
3′ UTR	2.9%	3.6%	<2.2×10^−16^	2.1%	4.3%	1.5×10^−9^
Intragenic	26.0%	24.6%	.003	31.4%	17.1%	<2.2×10^−16^

Note that each row in the table represents a Fisher’s exact test that tests for enrichment of associated or unassociated sites for the relevant category (e.g. CpG islands).

* NS indicates the p-value is not significant (p>.05).

Gene set enrichment analysis (GSEA) was used to gain further insight into the functional context of GA-associated CpG sites (FDR<.05; Table 4). Prominent biological processes that were enriched in GA-associated CpG sites were related to embryonic development. For example, 9 sites in the 5′UTR and body of histone deacetylase 4 (*HDAC4*, 1.3.x10^−11^<p<9.8×10^−4^; −.0023<Δβ per week<−.01) have higher methylation levels in fetuses with lower GA. *HDAC4* is involved in numerous identified pathways including system development and multicellular organismal development, anatomical structure development, organ development, and nervous system development. Several other CpG sites involved in epigenetic regulation during development were also identified. Specifically, CpG sites in the gene body of *DNMT1* (DNA methyltransferase 1; p = 3.4×10^−5^; t = −4.7; Δβ per week = −.0034), the gene body of *DNMT3A (*p = 6.7×10^−4^; t = −3.7; Δβ per week = −.0042*),* the 5′UTR of *DNMT3B* (4.5×10^−6^<p<8.4×10^−4^; 3.6<t<5.3;.0040<Δβ per week<.0053) and the 5′UTR of *TET1* (tet methylcytosine dioxygenase 1; 1.5×10^−7^<p<2.7×10^−4^; 4.0<t<6.4;.0046<Δβ per week<.01) also associate with GA (Table S1).

**Table 4:** Gene enrichment analysis of CpG sites that associated with GA (FDR<.05).

GO Term: Biological Processes	Size	NES	FDR q-value
Neuron differentiation	29	2.69	0.001
Generation of neurons	33	2.53	0.003
Multicellular organismal development	307	2.39	0.006
Anatomical structure development	309	2.32	0.007
System development	258	2.24	0.011
Neurogenesis	34	2.23	0.011
Cellular morphogensis during differentiation	16	2.17	0.014
Neuron development	20	2.09	0.023
Embryonic development	24	2.08	0.022
Organ development	171	2.04	0.026
Nervous system development	133	2	0.033
Skeletal development	34	1.95	0.042
Anatomical structure morphogenesis	122	1.9	0.048
Negative regulation of biological process	183	−2.18	0.043
Apoptosis GO	127	−2.17	0.036
Positive regulation of I-kappaB kinase NF-kappaB cascade	23	−2.13	0.042
**GO Term: Cellular Components**			
Extracellular region part	75	3.13	<.001
Extracellular region	97	3.02	<.001
Extracellular space	48	2.66	<.001
Proteinaceous extracellular matrix	26	2.24	0.009
Extracellular matrix	26	2.2	0.009
Extracellular matrix part	20	2.05	0.017
Intracellular organelle part	219	−2.12	0.042
Organelle part	219	−2.12	0.021
Nuclear part	102	−2.08	0.019
Microtubule cytoskeleton	37	−2.03	0.023
Nucleus	313	−2.02	0.02
Ribonucleoprotein complex	17	−1.97	0.026
Membrane enclosed lumen	73	−1.93	0.029
Cytoskeletal part	53	−1.93	0.025
Nuclear lumen	66	−1.92	0.023
Organelle lumen	73	−1.91	0.023
Cytoskeleton	105	−1.9	0.022
Nucleoplasm	56	−1.87	0.025
Nucleoplasm part	37	−1.79	0.038
Cell cortex	16	−1.74	0.048
Non membrane bound organelle	138	−1.73	0.047
Intracellular non membrane bound organelle	138	−1.73	0.046
Macromolecular complex	166	−1.71	0.049

NES is the normalized enrichment score.

Among the enriched cellular components are several groups that relate to extracellular regions. Remodeling of the extracellular matrix is required to support pregnancy and parturition [30] and increased attention has recently been focused on the role of matrix metallopeptidases (MMPs) and tissue inhibitors of metalloproteinases (TIMPs) in preterm birth [31]. In this study, 4 CpG sites in the promoter of *MMP9* (5.6×10^−7^<p<3.2×10^−4^; 4.0<t<6.0;.0021<Δβ per week<.0033) had higher methylation with increasing gestational age. *MMP9* is involved in the breakdown of the extracellular matrix in the process of cervical ripening, and increased expression has been seen in pPROM compared to preterm birth with intact membranes [32]. Further, 1 CpG site in the gene body of the MMP9 inhibitor, *TIMP2* also associates with GA (p = 1.4×10^−5^; t = −5.0; Δβ per week = −.0053).

To complement our discovery approach, we evaluated the association between CpG sites in genes that had been associated with GA in a previous study that used a less dense array with 27,578 CpG sites [18] (Table 5). 21 of 26 CpG sites (80.8%) significantly associated with GA in the previous study replicate in the present cohort (7.5×10^−11^<p<.05; −8.1<t<8.9; −.01<Δβ per week<.01). Notably, methylation of a CpG site in corticotrophin-releasing hormone binding protein (*CRHBP*) increased with decreasing GA (t = −4.49; p = 6.5×10^−5^; Δβ per week = .01). CRHBP regulates corticotrophin-releasing hormone (CRH), a principal regulator of the hypothalamic-pituitary-adrenal (HPA) axis. In addition, methylation increased in a CpG site in the promoter of *PIK3CD* (phosphatidylinositol-4,5-bisphosphate 3-kinase, catalytic subunit delta) with decreasing GA (p = 2.4×10^−8^; t = −7.0; Δβ per week = −.0062). The therapeutic value of PIK3CD inhibitors is currently being explored as anti-inflammatory drugs [33].

**Table 5:** Replication of CpG sites previously associated with GA [18].

Probe ID	Gene	Δβ per week	t-statistic	p-value
cg09523691	*ATG12*	−.0030	−3.33	.0020
cg16536918	*AVP*	−.0065	−3.08	.0039
cg25551168	*AVP*	−.0067	−3.61	8.7×10^−4^
cg01143454	*C20orf141*	−.0034	−1.66	.10
cg26799474	*CASP8*	−.0087	−4.03	2.6×10^−4^
cg13813391	*CMTM2*	−.0067	−3.99	2.9×10^−4^
cg21842274	*CRHBP*	−.014	−4.49	6.5×10^−5^
cg11540997	*DUOX2*	.0032	3.27	.0023
cg14409083	*EMP1*	−.0052	−3.00	.0047
cg15626350	*ESR1*	.011	3.83	4.6×10^−4^
cg20291222	*GLIPR1L2*	−.0065	−3.63	8.4×10^−4^
cg16098726	*GP9*	.0018	1.30	.20
cg14423778	*MBNL1*	−.0064	−2.96	.0052
cg05294455	*MYL4*	.010	4.83	2.3×10^−5^
cg26267561	*OXT*	−.0029	−1.43	.16
cg20994801	*PIK3CD*	−.0061	−7.01	2.4×10^−8^
cg15561986	*POMT2*	−.0029	−3.55	.0010
cg00594952	*RIMS3*	.0035	3.21	.0027
cg22417398	*SCYL1*	. −.0033	−4.48	6.7×10^−5^
cg10652277	*SLC30A9*	1.4×10^−4^	.58	.57
cg16301617	*TMC6*	. −.011	−6.86	3.8×10^−8^
cg26385222	*TMEM176B*	.0057	3.42	.0015
cg00411097	*TMEM184A*	−.0073	−5.63	1.8×10^−6^
cg27210390	*TOM1L1*	.0086	8.92	7.5×10^−11^
cg06051311	*TRIM15*	−.011	−8.08	8.8×10^−10^
cg09244244	*TTC37*	−.0025	−1.53	.13

Δβ per week represents the average increase in β-value associated with each additional week of GA.

One limitation of this strategy is that GA and PTB represent correlated but etiologically distinct phenotypes. Thus, replicating associations observed with GA may not capture the same breadth of candidate genes that could be explored in a study focused on PTB. For example, *IGFBP1* has been considered as a marker for preterm in vaginal infection and leaking amniotic fluid [34]. We observed associations between GA and 6 CpG sites in insulin-like growth factor 2 mRNA binding protein 1 *(IGF2BP1;* 2.1×10^−12^<p<1.9×10^−4^; −4.1<t<10.2; −.0087<Δβ per week<.02) located in the gene body though the direction of the association changed based on proximity of the CpG site to the CpG island.

## Discussion

By examining DNA methylation across the genome, we identified 29 CpG sites that associated with PTB independently of GA in leukocyte DNA from high-risk African American fetuses. Among these are CpG sites in *GSK3B* (glycogen synthase kinase 3 beta), which is involved in neuronal migration, development, and polarization, particularly during early embryonic development [35], [36]. Interestingly, *GSK3B* is a negative regulator of *MAML1 (*mastermind-like 1*)* [*37*], a component of the Notch pathway [38], [39], and a CpG site in *MAML1* also associated with PTB. *GSK3B* decreases transcription in the notch pathway through inhibition of *MAML1* [37]. Consistent with the role of *GSK3B* in regulating *MAML1*, there was an inverse relationship in the associations for the CpG sites in these genes. During development, the Notch pathway is integral to several developmental processes including neurogenesis, cardiovascular function, angiogenesis as well as intestinal and bone development [40] **.**

Additionally, 9637 CpG sites associated with GA when it was modeled separately from PTB. Our analyses suggest enrichment of GA-associated CpG sites in biological processes involved not only in embryonic and organ development but also in neurogenesis, nervous system development and neuron development. These processes involve extensive epigenetic regulation so it is not surprising that we observed associations with CpG sites in genes related to shaping epigenetic patterns during development: *HDAC4, DNMT1, DNMT3A, DNMT3B,* and *TET1*. For example, CpG sites in *TET1* and *DNMT3B* have lower DNA methylation in subjects with shorter GA. *TET1* functions to hydroxylate 5′methylcyctosine(mC) into 5′hydroxymethyl cytosine (hmC) [41]. *TET1* has been implicated is normal embryogenesis, and the depletion of *TET1* leads to low birth weight (LBW) in mouse pups [42]. *TET1* promotes active demethylation while *DNMT3B* promotes *de novo* methylation; these two processes are highly involved in the establishment of tissue-specific DNA methylation patterns during development [41], [43]. Though these results are indicative of the developmental time sampled (i.e. 32 versus 38 weeks), they may also support the hypothesis of epigenetic programming during fetal development [44].

The cellular components most enriched for genes with GA-associated CpG sites were primarily related to the extracellular region. Genes such as *MMP9* and *TIMP2* are integral to the process of parturition [45]. MMP9 has previously been considered as a biomarker for preterm birth [46] and has been thought to play a role in premature rupture of the membranes (PROM) because of its role in the degradation of the amniochorion basement membranes [47]. MMP9 levels are higher following PROM when compared to term deliveries, while TIMP2 levels decrease. DNA methylation differences in these and other genes related to extracellular matrix function support further study of the role of the fetal extracellular matrix throughout pregnancy and during parturition.

Many studies of fetal programming or prenatal exposures focus on fetuses with intrauterine growth restrictions or that were small for gestational age. Recent studies in the field support associations between GA and both DNA methylation and gene expression differences, but note lesser or no associations with birth weight [18], [48]. Similarly, in this study we identified numerous associations between DNA methylation and PTB, which is measured by GA, but no associations with percentile birth weight. Based on this, Stunkel and colleagues hypothesize that birth weight may be a less appropriate measure of adverse outcomes than GA [48]. Along these lines, we identified associations between GA and DNA methylation of CpG sites in insulin-like growth factor 2 mRNA binding protein 1 *(IGF2BP1),* a developmentally regulated gene that binds IGF2 and has been a focus of the fetal programming literature [49]. DNA methylation in IGF2 has been linked to various pregnancy-related conditions including birth weight [50]. IGFBP proteins are secreted from the placenta, decidua and fetal membranes in increasing amounts across gestation and are abundant in amniotic fluid [51]. Detection of IGFBP-1 in cervical–vaginal secretions is reliably used to detect preterm premature rupture of the membranes, which precedes 40% of spontaneous PTB cases [52], [53]. However, we were not able identify PTB-associated DNA methylation differences.

Our results were consistent with previous studies of DNA methylation in gestational age. Despite differences between cohorts and study design, we replicated >80% of CpG sites associated with GA in a previous study [18] further supporting the role of these genes in embryonic development and parturition. For example, CpG sites in *CRHBP* associated with GA. CHRBP binds CRH limiting its activity, and changes in the relative ratios of CRH to CRHBP associate with timing of birth [54], [55]. Prior to parturition, CRHBP levels decrease while CRH levels increase facilitating labor in both term and preterm deliveries [56]. In women who deliver preterm there is a decrease in plasma levels of CRHBP compared to women who deliver term [57].

The goal of this study was to identify associations between DNA methylation and PTB. However, PTB is defined by GA at birth; thus, the differences observed may correspond to differences in the developmental stage versus the causes or consequences of PTB. In this study, the correlation between association tests for PTB and GA is strong (r = .93; Figure S2), and delineation of these factors is complex, particularly in a study with a relatively small sample size. Thus, larger studies will be required to identify DNA methylation differences exclusive to PTB. Future studies of methylation as a risk factor for PTB should also focus on maternal methylation during pregnancy; a prospective study design could avoid confounding due to differences in GA by sampling at standardized time points, and could allow comparisons between maternal and fetal methylation changes. However, even with our relatively small sample of fetal cord blood DNA, we were able to identify robust associations using a stringent phenotype definition that compared samples from early preterm and later term deliveries in a high-risk cohort; in general, African American women are 3–4 times more likely than Caucasian women to deliver in the early preterm period [7]. Another limitation is the use of whole umbilical cord blood. While an ideal design would examine DNA methylation in a single cell type, this approach and our results were consistent with previous studies [18], [58]. Still, our results support the idea that epigenetic differences exist in fetuses born at different gestational ages. Recent studies suggest that DNA methylation patterns in many genes may be relatively stable over the first two years of life [16], [17], and further studies will be necessary to determine whether persisting differences in DNA methylation may underlie the physiological correlates of PTB.

## Supporting Information

Figure S1 **Scatter plots of the unmethylated vs. methylated signals (A versus B) for the five PTB-associated CpG sites that have 1000 Genomes SNPs within the probe.**
(TIF)

Figure S2 **Correlation between the t-statics depicting association analysis of CpG sites with PTB (x-axis) compared to GA (y-axis).** All CpG sites are depicted whether or not they were associated with the outcome. In order to compare more directly compare the results from analyses of PTB and GA, we reversed the sign of the t-statistics for PTB in this plot.
(TIF)

Table S1 CpG sites that associate with GA (FDR<.05).
(CSV)

## References

[1] Beck S, Wojdyla D, Say L, Betran AP, Merialdi M, et al (2010) The worldwide incidence of preterm birth: a systematic review of maternal mortality and morbidity. Bull World Health Organ 88: 31–38. 20428351 10.2471/BLT.08.062554 PMC2802437

[2] Yuan W, Basso O, Sorensen HT, Olsen J (2001) Indicators of fetal growth and infectious disease in childhood–a birth cohort with hospitalization as outcome. Eur J Epidemiol 17: 829–834. 12081101 10.1023/a:1015626329533

[3] Bhutta AT, Cleves MA, Casey PH, Cradock MM, Anand KJ (2002) Cognitive and behavioral outcomes of school-aged children who were born preterm: a meta-analysis. JAMA 288: 728–737. 12169077 10.1001/jama.288.6.728

[4] Loftin RW, Habli M, Snyder CC, Cormier CM, Lewis DF, et al (2010) Late preterm birth. Rev Obstet Gynecol 3: 10–19. 20508778 PMC2876317

[5] Nosarti C, Reichenberg A, Murray RM, Cnattingius S, Lambe MP, et al (2012) Preterm birth and psychiatric disorders in young adult life. Arch Gen Psychiatry 69: E1–8. 10.1001/archgenpsychiatry.2011.1374 22660967

[6] Behrman REB (2007) Preterm Birth: Causes, Consequences, and Prevention; Outcomes CoUPBaAH, editor. Washington, D.C.: The National Academies Press. 792 p. 20669423

[7] Goldenberg RL, Culhane JF, Iams JD, Romero R (2008) Epidemiology and causes of preterm birth. Lancet 371: 75–84. 18177778 10.1016/S0140-6736(08)60074-4 PMC7134569

[8] Menon R, Conneely KN, Smith AK (2012) DNA methylation: an epigenetic risk factor in preterm birth. Reprod Sci 19: 6–13. 22228737 10.1177/1933719111424446 PMC5933152

[9] Kyrklund-Blomberg NB, Granath F, Cnattingius S (2005) Maternal smoking and causes of very preterm birth. Acta Obstet Gynecol Scand 84: 572–577. 15901269 10.1111/j.0001-6349.2005.00848.x

[10] Jeffcoat MK, Geurs NC, Reddy MS, Goldenberg RL, Hauth JC (2001) Current evidence regarding periodontal disease as a risk factor in preterm birth. Ann Periodontol 6: 183–188. 11887462 10.1902/annals.2001.6.1.183

[11] Goldberg AD, Allis CD, Bernstein E (2007) Epigenetics: a landscape takes shape. Cell 128: 635–638. 17320500 10.1016/j.cell.2007.02.006

[12] Barker DJ (1990) The fetal and infant origins of adult disease. BMJ 301: 1111. 2252919 10.1136/bmj.301.6761.1111 PMC1664286

[13] Barker DJ, Gelow J, Thornburg K, Osmond C, Kajantie E, et al (2010) The early origins of chronic heart failure: impaired placental growth and initiation of insulin resistance in childhood. Eur J Heart Fail 12: 819–825. 20504866 10.1093/eurjhf/hfq069 PMC5477852

[14] Paulsen M, Ferguson-Smith AC (2001) DNA methylation in genomic imprinting, development, and disease. J Pathol 195: 97–110. 11568896 10.1002/path.890

[15] Ginder GD, Gnanapragasam MN, Mian OY (2008) The role of the epigenetic signal, DNA methylation, in gene regulation during erythroid development. Curr Top Dev Biol 82: 85–116. 18282518 10.1016/S0070-2153(07)00004-X

[16] Wang D, Liu X, Zhou Y, Xie H, Hong X, et al (2012) Individual variation and longitudinal pattern of genome-wide DNA methylation from birth to the first two years of life. Epigenetics 7: 594–605. 22522910 10.4161/epi.20117 PMC3398988

[17] Beyan H, Down TA, Ramagopalan SV, Uvebrant K, Nilsson A, et al (2012) Guthrie card methylomics identifies temporally stable epialleles that are present at birth in humans. Genome Res 22: 2138–2145. 22919074 10.1101/gr.134304.111 PMC3483543

[18] Schroeder JW, Conneely KN, Cubells JC, Kilaru V, Newport DJ, et al (2012) Neonatal DNA methylation patterns associate with gestational age. Epigenetics 6: 1498–1504. 10.4161/epi.6.12.18296 PMC3256334 22139580

[19] Pan H, Chen L, Dogra S, Teh AL, Tan JH, et al (2012) Measuring the methylome in clinical samples: improved processing of the Infinium Human Methylation450 BeadChip Array. Epigenetics 7: 1173–1187. 22964528 10.4161/epi.22102 PMC3469459

[20] Roessler J, Ammerpohl O, Gutwein J, Hasemeier B, Anwar SL, et al (2012) Quantitative cross-validation and content analysis of the 450k DNA methylation array from Illumina, Inc. BMC Res Notes 5: 210. 22546179 10.1186/1756-0500-5-210 PMC3420245

[21] Barfield RT, Kilaru V, Smith AK, Conneely KN (2012) CpGassoc: an R function for analysis of DNA methylation microarray data. Bioinformatics 28: 1280–1281. 22451269 10.1093/bioinformatics/bts124 PMC3577110

[22] Kilaru V, Barfield RT, Schroeder JW, Smith AK, Conneely KN (2012) MethLAB: a graphical user interface package for the analysis of array-based DNA methylation data. Epigenetics 7: 225–229. 22430798 10.4161/epi.7.3.19284 PMC3335946

[23] Oken E, Kleinman KP, Rich-Edwards J, Gillman MW (2003) A nearly continuous measure of birth weight for gestational age using a United States national reference. BMC Pediatr 3: 6. 12848901 10.1186/1471-2431-3-6 PMC169185

[24] Du P, Zhang X, Huang CC, Jafari N, Kibbe WA, et al (2010) Comparison of Beta-value and M-value methods for quantifying methylation levels by microarray analysis. BMC Bioinformatics 11: 587. 21118553 10.1186/1471-2105-11-587 PMC3012676

[25] Feinberg AP (2007) Phenotypic plasticity and the epigenetics of human disease. Nature 447: 433–440. 17522677 10.1038/nature05919

[26] Irizarry RA, Ladd-Acosta C, Wen B, Wu Z, Montano C, et al (2009) The human colon cancer methylome shows similar hypo- and hypermethylation at conserved tissue-specific CpG island shores. Nat Genet 41: 178–186. 19151715 10.1038/ng.298 PMC2729128

[27] Subramanian A, Tamayo P, Mootha VK, Mukherjee S, Ebert BL, et al (2005) Gene set enrichment analysis: a knowledge-based approach for interpreting genome-wide expression profiles. Proc Natl Acad Sci U S A 102: 15545–15550. 16199517 10.1073/pnas.0506580102 PMC1239896

[28] Mootha VK, Lindgren CM, Eriksson KF, Subramanian A, Sihag S, et al (2003) PGC-1alpha-responsive genes involved in oxidative phosphorylation are coordinately downregulated in human diabetes. Nat Genet 34: 267–273. 12808457 10.1038/ng1180

[29] Alisch RS, Barwick BG, Chopra P, Myrick LK, Satten GA, et al (2012) Age-associated DNA methylation in pediatric populations. Genome Res 22: 623–632. 22300631 10.1101/gr.125187.111 PMC3317145

[30] Weiss A, Goldman S, Shalev E (2007) The matrix metalloproteinases (MMPS) in the decidua and fetal membranes. Front Biosci 12: 649–659. 17127325 10.2741/2089

[31] Tency I, Verstraelen H, Kroes I, Holtappels G, Verhasselt B, et al (2012) Imbalances between matrix metalloproteinases (MMPs) and tissue inhibitor of metalloproteinases (TIMPs) in maternal serum during preterm labor. PLoS One 7: e49042. 23145060 10.1371/journal.pone.0049042 PMC3493509

[32] Romero R, Chaiworapongsa T, Espinoza J, Gomez R, Yoon BH, et al (2002) Fetal plasma MMP-9 concentrations are elevated in preterm premature rupture of the membranes. Am J Obstet Gynecol 187: 1125–1130. 12439489 10.1067/mob.2002.127312

[33] Harris SJ, Foster JG, Ward SG (2009) PI3K isoforms as drug targets in inflammatory diseases: lessons from pharmacological and genetic strategies. Curr Opin Investig Drugs 10: 1151–1162. 19876783

[34] Vogel I, Gronbaek H, Thorsen P, Flyvbjerg A (2004) Insulin-like growth factor binding protein 1 (IGFBP-1) in vaginal fluid in pregnancy. In Vivo 18: 37–41. 15011749

[35] Aparicio IM, Garcia-Herreros M, Fair T, Lonergan P (2010) Identification and regulation of glycogen synthase kinase-3 during bovine embryo development. Reproduction 140: 83–92. 20427566 10.1530/REP-10-0040

[36] Hur EM, Zhou FQ (2010) GSK3 signalling in neural development. Nat Rev Neurosci 11: 539–551. 20648061 10.1038/nrn2870 PMC3533361

[37] Saint Just Ribeiro M, Hansson ML, Lindberg MJ, Popko-Scibor AE, Wallberg AE (2009) GSK3beta is a negative regulator of the transcriptional coactivator MAML1. Nucleic Acids Res 37: 6691–6700. 19740771 10.1093/nar/gkp724 PMC2777432

[38] Watanabe T, Oyama T, Asada M, Harada D, Ito Y, et al (2013) MAML1 Enhances the Transcriptional Activity of Runx2 and Plays a Role in Bone Development. PLoS Genet 9: e1003132. 23326237 10.1371/journal.pgen.1003132 PMC3542067

[39] Shen H, McElhinny AS, Cao Y, Gao P, Liu J, et al (2006) The Notch coactivator, MAML1, functions as a novel coactivator for MEF2C-mediated transcription and is required for normal myogenesis. Genes Dev 20: 675–688. 16510869 10.1101/gad.1383706 PMC1413284

[40] Alva JA, Iruela-Arispe ML (2004) Notch signaling in vascular morphogenesis. Curr Opin Hematol 11: 278–283. 15314528 10.1097/01.moh.0000130309.44976.ad

[41] Williams K, Christensen J, Pedersen MT, Johansen JV, Cloos PA, et al (2011) TET1 and hydroxymethylcytosine in transcription and DNA methylation fidelity. Nature 473: 343–348. 21490601 10.1038/nature10066 PMC3408592

[42] Dawlaty MM, Ganz K, Powell BE, Hu YC, Markoulaki S, et al (2011) Tet1 is dispensable for maintaining pluripotency and its loss is compatible with embryonic and postnatal development. Cell Stem Cell 9: 166–175. 21816367 10.1016/j.stem.2011.07.010 PMC3154739

[43] Okano M, Bell DW, Haber DA, Li E (1999) DNA methyltransferases Dnmt3a and Dnmt3b are essential for de novo methylation and mammalian development. Cell 99: 247–257. 10555141 10.1016/s0092-8674(00)81656-6

[44] Hogg K, Price EM, Hanna CW, Robinson WP (2012) Prenatal and perinatal environmental influences on the human fetal and placental epigenome. Clin Pharmacol Ther 92: 716–726. 23047650 10.1038/clpt.2012.141

[45] Maymon E, Romero R, Pacora P, Gervasi MT, Gomez R, et al (2000) Evidence of in vivo differential bioavailability of the active forms of matrix metalloproteinases 9 and 2 in parturition, spontaneous rupture of membranes, and intra-amniotic infection. Am J Obstet Gynecol 183: 887–894. 11035332 10.1067/mob.2000.108878

[46] Botsis D, Makrakis E, Papagianni V, Kouskouni E, Grigoriou O, et al (2006) The value of cervical length and plasma proMMP-9 levels for the prediction of preterm delivery in pregnant women presenting with threatened preterm labor. Eur J Obstet Gynecol Reprod Biol 128: 108–112. 16314025 10.1016/j.ejogrb.2005.10.022

[47] Fortunato SJ, Menon R, Lombardi SJ (1999) MMP/TIMP imbalance in amniotic fluid during PROM: an indirect support for endogenous pathway to membrane rupture. J Perinat Med 27: 362–368. 10642956 10.1515/JPM.1999.049

[48] Stunkel W, Pan H, Chew SB, Tng E, Tan JH, et al (2012) Transcriptome changes affecting Hedgehog and cytokine signalling in the umbilical cord: implications for disease risk. PLoS One 7: e39744. 22808055 10.1371/journal.pone.0039744 PMC3393728

[49] Perkins E, Murphy SK, Murtha AP, Schildkraut J, Jirtle RL, et al (2012) Insulin-like growth factor 2/H19 methylation at birth and risk of overweight and obesity in children. J Pediatr 161: 31–39. 22341586 10.1016/j.jpeds.2012.01.015 PMC3360130

[50] Hoyo C, Fortner K, Murtha AP, Schildkraut JM, Soubry A, et al (2012) Association of cord blood methylation fractions at imprinted insulin-like growth factor 2 (IGF2), plasma IGF2, and birth weight. Cancer Causes Control 23: 635–645. 22392079 10.1007/s10552-012-9932-y PMC3313040

[51] Martina NA, Kim E, Chitkara U, Wathen NC, Chard T, et al (1997) Gestational age-dependent expression of insulin-like growth factor-binding protein-1 (IGFBP-1) phosphoisoforms in human extraembryonic cavities, maternal serum, and decidua suggests decidua as the primary source of IGFBP-1 in these fluids during early pregnancy. J Clin Endocrinol Metab 82: 1894–1898. 9177402 10.1210/jcem.82.6.3974

[52] Lockwood CJ, Wein R, Chien D, Ghidini A, Alvarez M, et al (1994) Fetal membrane rupture is associated with the presence of insulin-like growth factor-binding protein-1 in vaginal secretions. Am J Obstet Gynecol 171: 146–150. 7518190 10.1016/0002-9378(94)90461-8

[53] Rutanen EM, Karkkainen TH, Lehtovirta J, Uotila JT, Hinkula MK, et al (1996) Evaluation of a rapid strip test for insulin-like growth factor binding protein-1 in the diagnosis of ruptured fetal membranes. Clin Chim Acta 253: 91–101. 8879841 10.1016/0009-8981(96)80001-e

[54] Hillhouse EW, Grammatopoulos DK (2002) Role of stress peptides during human pregnancy and labour. Reproduction 124: 323–329. 12201805 10.1530/rep.0.1240323

[55] McLean M, Bisits A, Davies J, Woods R, Lowry P, et al (1995) A placental clock controlling the length of human pregnancy. Nat Med 1: 460–463. 7585095 10.1038/nm0595-460

[56] Hobel CJ, Arora CP, Korst LM (1999) Corticotrophin-releasing hormone and CRH-binding protein. Differences between patients at risk for preterm birth and hypertension. Ann N Y Acad Sci 897: 54–65. 10676435 10.1111/j.1749-6632.1999.tb07878.x

[57] Perkins AV, Eben F, Wolfe CD, Schulte HM, Linton EA (1993) Plasma measurements of corticotrophin-releasing hormone-binding protein in normal and abnormal human pregnancy. J Endocrinol 138: 149–157. 7852886 10.1677/joe.0.1380149

[58] Adkins RM, Krushkal J, Tylavsky FA, Thomas F (2011) Racial differences in gene-specific DNA methylation levels are present at birth. Birth Defects Res A Clin Mol Teratol 91: 728–736. 21308978 10.1002/bdra.20770 PMC3429933

