# Supplementary figures and images for: Fetal DNA Methylation Associates with Early Spontaneous Preterm Birth and Gestational Age
Source: PLoS One. 2013 Jun 27;8(6):e67489. doi: 10.1371/journal.pone.0067489 (PMC3694903; doi:10.1371/journal.pone.0067489)

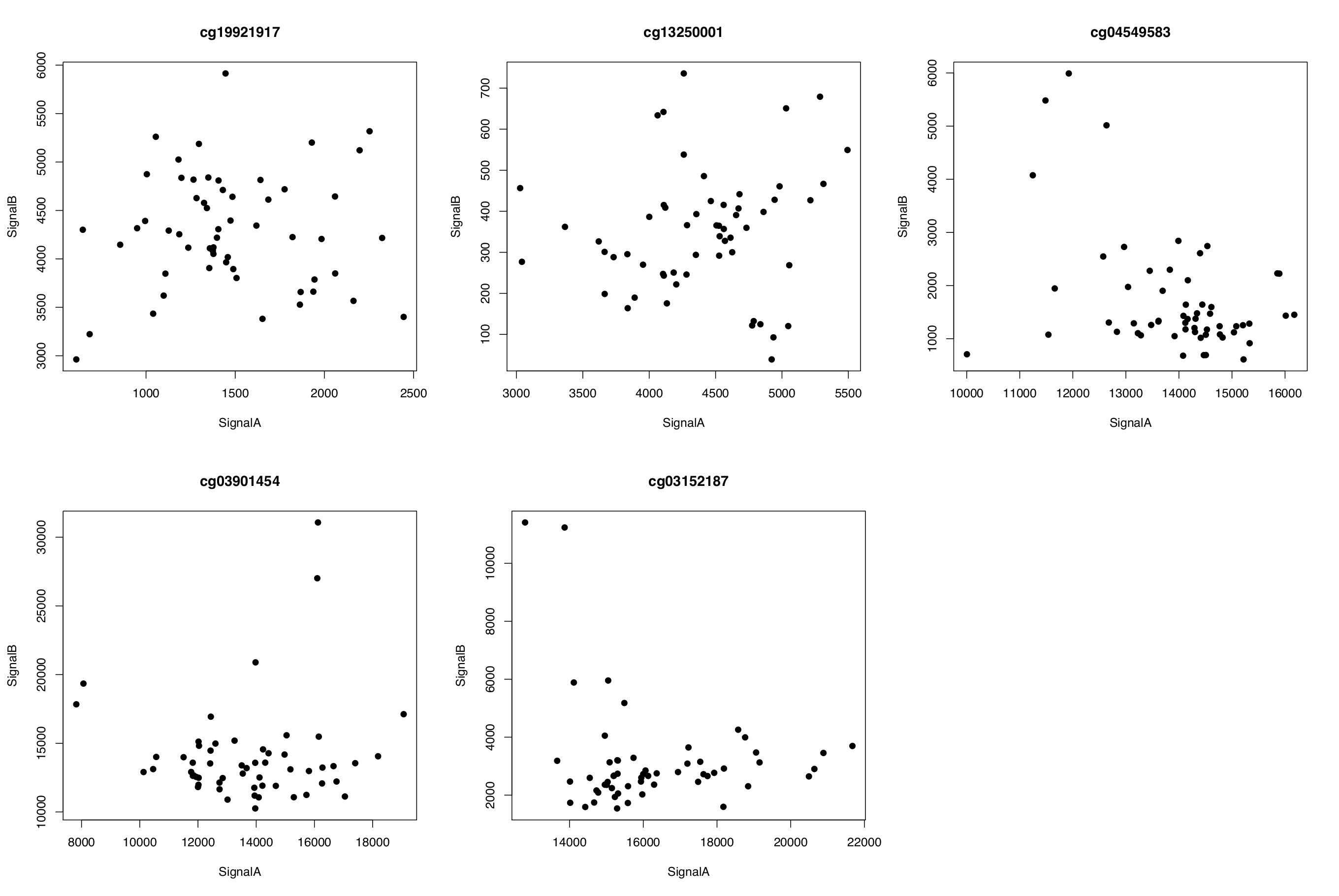

Supplement: Figure S1 — Scatter plots of the unmethylated vs. methylated signals (A versus B) for the five PTB-associated CpG sites that have 1000 Genomes SNPs within the probe. (TIF) [file pone.0067489.s001.tif]
